# Early adolescents’ physical activity and nutrition beliefs and behaviours

**DOI:** 10.1080/17482631.2022.2050523

**Published:** 2022-03-15

**Authors:** Miranda Hawks, Angela Bratton, Sandra Mobley, Vernon Barnes, Steven Weiss, Julie Zadinsky

**Affiliations:** a Byrdine F. Lewis College of Nursing and Health Professions, Georgia State University, Atlanta, Georgia, USA; b Katherine Reese Pamplin College of Arts, Humanities, and Social Sciences, Augusta University, Augusta, Georgia, USA; c Medical College of Georgia, Augusta University, Augusta, Georgia, USA; d College of Nursing, Augusta University, Augusta, Georgia, USA

**Keywords:** Adolescence, community organization, early adolescents, ethnography, healthy eating, interviews, physical activity, qualitative, nutrition, southeastern USA

## Abstract

Obesity in early adolescents is a public health concern and presents a risk for poor health outcomes later in life. Early adolescents’ beliefs and behaviours of physical activity and healthy eating are not well understood. The purpose of this ethnographic inquiry was to explore the physical activity and healthy eating beliefs and behaviours of early adolescents (n = 15) who attended a community organization in an urban cluster in the Southeastern USA. Data from semi-structured interviews, participant observations and artefact collection were analysed using constant comparative analysis. We learned that five main influences on the early adolescents’ healthy eating and physical activity included recognizing benefits of physical activity and healthy eating, family influences, connecting with the community, peer influences, and developing a sense of self. Findings highlight community influences on early adolescents’ physical activity and healthy eating beliefs and behaviours that should be taken into account when developing health promotion strategies.

## Background

Obesity during early adolescence is a significant public health problem that has a number of adverse consequences such as increasing the risk of developing Type 2 Diabetes, hypertension, atherosclerosis and premature cardiovascular disease (Kumar & Kelly, 2017; McPhee et al., 2020). From 1975 to 2016, the worldwide adolescent age standardized prevalence of obesity increased from 0.7% to 5.6% in girls and from 0.9% to 7.8% in boys (Abarca-Gómez et al., 2017). Additionally, obesity rates ranged from 15% to 19.9% in the southeastern region of the USA (US) in 2019 to 2020 (Robert Wood Johnson Foundation, 2021). In 2017, the region in which the study was conducted ranked 17th in the US for the prevalence of childhood obesity (Satcher, 2017). Moreover, higher population density schools in the southeastern region of the US have been associated with healthier Body Mass Indexes (BMI) and increased aerobic capacity (Bai, Saint-Maurice & Welk, 2017).

Studies have shown that the interaction of environmental, nutritional, and physical activity factors contribute to obesity during early adolescence (Kumar & Kelly, 2017). Also, maintaining healthy physical activity and nutrition practices in this age group is essential for preventing obesity later in life (World Health Organization, 2020). Moreover, it has become increasingly more challenging for early adolescents to adopt and maintain health-promoting physical activity and nutrition practices due to challenges in their individual and interpersonal environment (Banna et al., 2015; Kebbe et al., 2017). Quantitative studies have investigated social and ecological factors contributing to obesity in this population (Kim & Kim, 2019). However, there is a gap in the literature related to qualitative research designed to investigate environmental factors and social contributors to obesity from an ecological perspective using ethnography in this age group (Waters et al., 2011).

Early adolescence, which includes adolescents 10 to 14 years of age, is the first developmental stage in adolescence and is marked by the onset of puberty and increased growth (Allen & Waterman, 2019). Social adaptation and development in early adolescents occur in school, at home, and in community organizations. These are important settings in which health beliefs and behaviours are formed. A study of early adolescents as they function in these settings on a daily basis can facilitate a better understanding of the beliefs and behaviours that initiate, facilitate, and inhibit healthy physical activity and nutrition practices.

The foregoing challenges highlight the need for qualitative research that investigates both physical activity and nutrition beliefs and behaviours in the cultural context of early adolescents’ environment. Community healthcare professionals need more information about early adolescents’ patterns of physical activity and nutrition beliefs and behaviours to inform health promotion strategies for this age group. Therefore, this ethnographic study was conducted to explore the physical activity and nutrition beliefs and behaviours of early adolescents attending a community organization in an urban cluster in the southeastern region of the US.

## Methods

### Design

**Figure 1:**
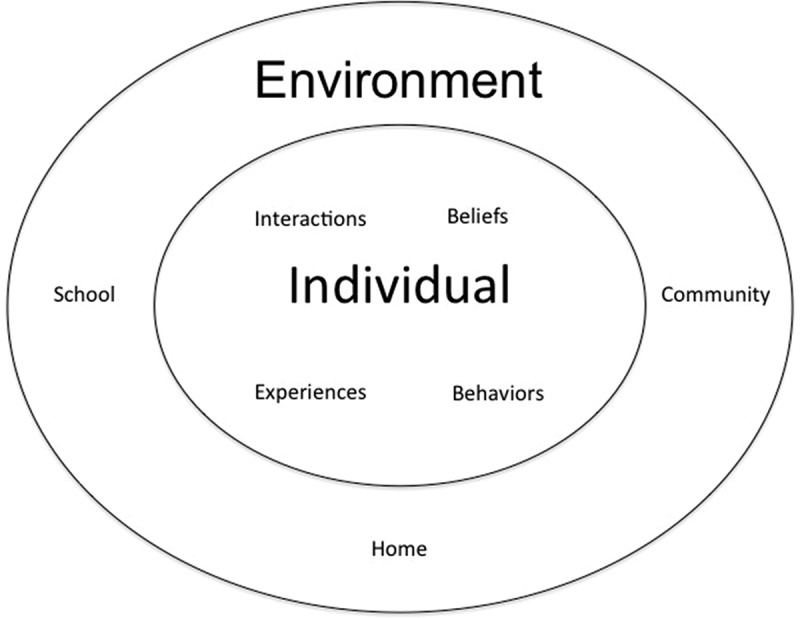
The conceptual framework of the individual and environment, which was developed from the description of Bronfenbrenner’s ecology model (Bronfenbrenner, 1994)

Participant observation, semi-structured interviews, and artefact collection were used to examine the participants’ beliefs and behaviours related to physical activity and healthy eating. The conceptual framework for this ethnographic study was derived from the concepts of individuals and their environment as defined in Bronfenbrenner’s, 1994 ecology model (see Figure 1), with a focus on facilitators and barriers of physical activity and healthy eating in early adolescents’ home and community environments. At the individual level, early adolescents’ roles, activities, and relationships were examined to better understand their experiences and interactions related to physical activity and healthy eating. At the environmental level, early adolescents’ roles, activities, and relationships were contextualized by examining their everyday lives in the community organization.

The concept of the individual is nested within the concept of the environment. Consideration of the concept of the environment contextualizes the concept of the individual. To provide reliable conclusions, the essential meaning of human action should be examined in the context of the social structure (i.e., immediate environment) in which it is generated (Bronfenbrenner, 1994).

Early adolescents’ interactions and relationships with other individuals (e.g., other early adolescents, parents, and teachers), objects (e.g., food items, physical activity equipment), and symbols (e.g., food-associated branding logos) were more easily understood and organized using a conceptual framework. Initially, the researchers focused the observations on early adolescents’ recreational activities, relationships with peers, and roles at home, school, and within the community to understand their behaviours regarding physical activity and nutrition. The researchers examined other people, objects, and circumstances that influenced the early adolescents’ interactions and behaviours as they occurred within their immediate environment in the community organization. The researchers also compared their examination of the factors that influenced adolescents’ behaviour with their stated beliefs regarding physical activity and nutrition to provide a more comprehensive understanding of the roles, activities, and relationships related to physical activity and nutrition. Early adolescents’ physical activity and nutrition beliefs and behaviours were better understood by examining the observations of adolescents’ interactions and behaviours in their environment together with their beliefs and experiences described during their interviews.

### Setting

The total population of the community where the community organization is located was 6,778 individuals in the last census taken before the research was conducted (U.S. Census Bureau, 2010). Approximately 63% of the total population was African American, and 35% was White (U.S. Census Bureau, 2010). Individuals of Hispanic or Latino origin comprised 1.5% of the total population (U.S. Census Bureau, 2010). There were slightly more females (57.1%) than males (42.9%) in the community, and approximately 20% of the individuals were 5 to 17 years of age (U.S. Census Bureau, 2010). The median household income averaged $24,347 per year in the community, which was approximately half of the average annual household income for other residents in the state where the research was conducted (U.S. Census Bureau, 2010). Furthermore, 30% of the community’s residents were considered to be living in poverty (U.S. Census Bureau, 2010). There was one middle school located in the community. Approximately 48% of the total middle school population was African American, and 48% was Caucasian (The Governor’s Office of Student Acheivement, 2011).

### Participants

Participants were recruited at a community organization in an urban cluster in the southeastern US using purposive and snowball sampling. This organization served children and adolescents aged 6 to18 years with programmes aimed at promoting health as well as social, educational, vocational, and character development. Adolescents 10 to 14 years of age at enrolment who were members of the community organization and had resided in the community for at least one year were eligible to participate in the study. Adults, including parents and teachers, were excluded from the study and the interview process. Participants were encouraged to discuss the study with others at the community organization who they thought might be interested in talking about physical activity and healthy eating. Fifteen early adolescents in the community organization participated in the study. One male early adolescent withdrew from the study after completing the first interview. However, the data from the one male adolescent who withdrew early were included in the data analysis. Heindicated that he did not have time to participate due to after-school sports activities. Sample characteristics are described in Table I.

**Table I.** Sample characteristics

Characteristic	Male Female	
	*n*	*n*
Race		
Black or African American	7	7
Mixed Hispanic and White	0	1
Age in years		
10	1	2
11	1	4
12	0	0
13	3	2
14	2	0

Note. This table illustrates the age, gender, and self-identified race of early adolescents in the study.

Approval was received from the university Institutional Review Board (IRB) and the community organization before starting study recruitment and enrolment. After these approvals had been obtained, the director of the community organization introduced the primary researcher to the adolescents and their parents. We established individual contact with adolescents’ parents when they were dropping off and picking up their adolescents at the organization for the winter after-school programme. At this time, we gave them the parent permission and assent forms that they could review to learn more about the study. At a time convenient to the parents, we conducted parent/adolescent consent meetings in a private room at the community organization with each early adolescent and one of their parents. During these meetings, we explained the study, answered any questions, allowed as much time as needed to consider study participation, and helped the parents and adolescents complete the parent permission and assent forms. Participants were recruited until data saturation was reached and no new themes were identified.

### Data collection

Three data collection methods—participant observation, semi-structured interviews, and artefact collection—were used to examine early adolescents’ physical activity and healthy eating beliefs and behaviours. Observations were made over a period of five months during the community organization’s winter program and for two weeks during the beginning of the summer program. Semi-structured interviews were conducted in the community organization’s private activity room with large windows and age-appropriate table and chairs. Three semi-structured interviews were conducted with each early adolescent using an interview schedule. The participants were asked during the interviews about the personal meaning that physical activity and nutrition held in their daily lives. Additionally, the participants were asked about their preferences and behaviours related to physical activity and nutrition. Information about the sources of physical activity and nutrition in the participants’ local community, community organization, and home was collected during the interviews. Furthermore, the interview guide was designed to identify any barriers to physical activity and healthy eating. The first and second interviews focused on questions related to identifying early adolescents’ physical activity and healthy eating beliefs and practices. The third interview was used to follow-up with early adolescents about questions, concepts, and themes identified during the researchers’ review of the first and second interview transcripts, to include commonalities and differences noted among the interviews of different adolescents.

The first two interviews lasted from 30 minutes to one and a half hours, and the third interview lasted approximately 30–45 minutes. All interviews were audio-recorded with a handheld device and were transcribed verbatim. Artefacts collected in the community included photographs as well as brochures and other publicly available materials related to physical activity and nutrition. Additionally, interview questions that could not be addressed during the first and second interviews were discussed during the third interview. Early adolescents’ stated beliefs conveyed during their first and second interviews were compared with their behaviours that the researchers had observed in the field. During the third interviews, the researchers addressed discrepancies between adolescents’ stated beliefs and observed behaviours during participant observation.

A focus of the third interview also included informal member checking. The researchers conducted informal member checking with all fourteen early adolescents who completed a third interview. Prior to the official reporting of the findings, the researchers presented the analysed data and conclusions to a few of the early adolescents for verification. The researchers conducted informal member checking during fieldwork throughout the data collection process. The researchers asked the early adolescents to confirm the findings as they were collected. If any of the early adolescents disagreed with the findings, the researchers collaborated with the early adolescents to understand how and why the disagreement occurred. The researchers also consulted with the study team members regarding any discrepancies in the findings.

### Data analysis

The interviews and field notes were analysed using constant comparative analysis (LeCompte & Schensul, 2010). Constant comparative analysis provided a method of analysing the data that was a reflexive and recursive process using discussions between the researcher and early adolescents to negotiate the meaning of early adolescents’ realities in the context of the conceptual model. NVivo (Version 10) software for data management was used to identify descriptive codes from the text, track data coding, and document the development and conceptualization of themes. Codes were identified that related to physical activity and healthy eating beliefs and behaviours at the level of the individual and the environment as defined by Bronfenbrenner’s (1993/1994) model. Sample codes and their definitions from the codebook for the interview data are presented in Table II. Tracking and documenting data analysis in this way produced an in-depth description of the data analysis process, which served as an audit trail.

**Table II.** Sample codes connected to early adolescents’ physical activity and nutrition beliefs and behaviours

Code	Definition
Benefits of healthy eating	Early adolescents’ beliefs and behaviours about how healthy eating is beneficial to them.
Benefits of physical activity	Early adolescents’ beliefs and behaviours about how physical activity is beneficial to them.
Community healthy eating beliefs	Early adolescents’ perception of their community’s beliefs about healthy eating.
Community physical activity beliefs	Early adolescents’ perception of their community’s beliefs about physical activity.
Effect of sensory perception on healthy eating	How the early adolescents described that taste, smell, and appearance of healthy foods influenced their healthy eating behaviours.
Family healthy eating beliefs	Early adolescents’ perception of their families’ beliefs about healthy eating.
Healthy eating at the community organization	The types and quantities of healthy foods that early adolescents reported and that the researcher observed them consuming at the community organization.
Healthy eating barriers in the community	A person, situation, or object that makes starting, continuing, or reinitiating healthy eating difficult or impossible for early adolescents in their community.
Healthy eating behaviours in the community	The daily routine and activities involved in early adolescents’ healthy eating in their community.
Healthy eating facilitators at home	A person, situation, or object that makes starting, continuing, or reinitiating healthy eating easier for early adolescents at home.
Healthy eating in the community	Early adolescents’ perception of healthy eating and features of the community related to healthy eating.
Healthy eating information in the community	Information about healthy eating in their community that early adolescents reported.
Healthy eating preferences	The types of healthy eating that early adolescents preferred.
Healthy eating knowledge	The knowledge that early adolescents had about healthy eating.
Personal healthy eating beliefs	Early adolescents’ individual beliefs about healthy eating.
Personal meaning of healthy eating	The meaning that healthy eating had to early adolescents with respect to important aspects of their lives, such as family and health.
Personal meaning of physical activity	The meaning that physical activity had to early adolescents in important aspects of their lives, such as family and health.
Personal physical activity beliefs	Early adolescents’ individual beliefs about physical activity.
Physical activity at home	Physical activities that early adolescents reported doing at home.
Physical activity barriers in the community	A person, situation, or object in or connected with the community that makes starting, continuing, or reinitiating physical activities difficult or impossible for early adolescents.
Physical activity facilitators at home	A person, situation, or object at home that makes starting, continuing, or reinitiating physical activities easier for early adolescents.
Physical activity in the community	Early adolescents’ perception of physical activity and features of the community related to physical activity.
Physical activity information at home	Information about physical activity at home that early adolescents reported.
Physical activity knowledge	The knowledge that early adolescents had about physical activity.
Physical activity preferences	The types of physical activity that early adolescents preferred.

Trustworthiness was enhanced through prolonged engagement with the digital recordings and transcripts of the interviews; triangulation of multiple data sources, to include interviews, participant observations, and artefacts; creation of an audit trail; and member checking. Member checking was useful in clarifying early adolescents’ beliefs and confirming emerging themes. Additionally, an inquiry and confirmability audit of the data collection and analysis process determined that the collected data sufficiently supported the findings.

### Ethical considerations

The study was approved by the university Social/Behavioural IRB [690,769–5] in January 2015. The research conformed to the ethical principles for medical research on human beings set out in the Declaration of Helsinki. The research study was guided by the ethical principles of autonomy, beneficence, non-maleficence, and justice, and all requirements for informed consent, confidentiality, and participant safety were met. Participation and data collection were confidentially conducted after parental permission and adolescent assent were obtained. The parents and adolescents who participated in the study received both verbal and written information about the study during the informed cosent process and were reminded throughout the study that they could withdraw from the study at any time without an explanation.

## Results

Our qualitative analysis identified five themes—recognizing benefits of physical activity and healthy eating, family influences, connecting with the community, peer influences, and developing a sense of self. Three core themes—family influences, connecting with the community, and peer influences—fit within the family and community levels of Bronfenbrenner's (1993/1994) model. Below we describe all five themes.

### Recognizing benefits of physical activity and healthy eating

All fifteen early adolescents recognized physical activity and healthy eating as beneficial for promoting their health and improving the quality of their lives. Early adolescents described their connections with individuals in their home, school, and the community organization as positive influences for learning about physical activity and healthy eating. They identified their mothers, grandmothers, and aunts as the main influence to eat healthy and be physically active. In addition, they reported that they learned about the benefits of physical activity and healthy eating from teachers in their health classes, coaches in their organized sports and physical education courses, and the director and staff at the community organization.

Early adolescents described specific benefits of physical activity and healthy eating associated with increases in physical and mental health. These benefits included increases in bone health, immune function, energy, weight loss, physical strength, positive mental attitude, and overall health. Most early adolescents also perceived physical activity and healthy eating as beneficial for their personal growth later in life.

All of the early adolescents described physical activity as a way for them to connect with their friends and family at home, school, and the community organization through “play” and other “fun” activities. They also were observed connecting with their peers at the community organization during physical activities such as basketball, dancing, and games. Participation in organized sports was one of the main forms of physical activity they used to connect with their peers.

Most of the early adolescents recognized physical activity as beneficial for preparing them for their current or anticipated participation in recreational or school organized sports. Being physically fit and eating healthy foods to prepare for participation in basketball was important to male early adolescents. Four of the seven male adolescents perceived playing basketball as beneficial for reaching the goal of becoming a professional basketball player as an adult. One of the male adolescents who wanted to become a professional basketball player described increased cardiovascular function as a unique benefit of physical activity.

All but one of eight female adolescents perceived physical activity as beneficial for weight maintenance or weight loss. Female adolescents learned about the importance of physical activity and healthy eating for weight loss from their mothers, grandmothers, and aunts, who seemed to think weight loss was important for increasing physical attractiveness more than for health promotion. One of these female early adolescents indicated that their peers also viewed physical activity as beneficial for weight loss to avoid bullying from other peers. Participant observations confirmed that bullying occurred at the community organization. One early adolescent articulated her association of being overweight or obese with bullying:

If you want to lose some weight, you can run, jump and do all kinds of healthy stuff. Because some people want to lose weight because other people been picking on them about how their size is. When people pick on them, they’re like they need to run the track and lose some weight and stuff.

All but one early adolescent perceived healthy eating as beneficial for increasing their energy so they could connect with others and their environments during physical activity. One female adolescent said, “it takes a lot of activeness to do dance and stuff. So, if you really don’t eat your vegetables, and you just load up on junk food it’s not going to help.” A male adolescent also clearly described the benefits of healthy eating: “When we’re playing basketball or some other sport, and they’re making up new games for us to play, it helps you get more used to running around and helps you get tired less easy.”

Early adolescents associated increased junk food consumption with decreased energy levels necessary for physical activity and health. Four early adolescents described the benefits of healthy eating through discussions about decreased consumption of junk food (e.g., donuts) to reduce negative health complications. Six early adolescents stated that if they ate increased quantities of junk food, they would sit around watching television or not have the required energy to go outdoors to play. One early adolescent clearly articulated the lack of energy they felt after eating junk food:

You can’t eat junk food all the time because you’ll be [laughter] … well, I’m not saying you’re going to be mentally slow, but you’ll physically be slow. Your body will weigh down, and you really won’t feel like doing anything but sitting down and eating. And you really won’t want to go anywhere, like walking or running or jogging, or going anywhere to be outside. You’ll mostly just sit on the couch and eat some food and watch TV all day. You won’t want to do anything.

Three male adolescents provided general descriptions of how healthy eating improved their quality of life. One of these early adolescents referenced healthy eating as beneficial for ensuring that their “life will be good” and as increasing their likelihood of trying new experiences. Another male adolescent referenced healthy eating as beneficial to helping them “stay on track.” Two of these male adolescents described healthy eating as being related to a prolonged lifespan and an increase in dental health (e.g., decreased number of dental caries).

### Family influences

*Family Influences* refers to early adolescents’ perceptions of the influences, both positive and negative, that families had on their physical activity and healthy eating beliefs and behaviours. Family included early adolescents’ mothers, fathers, siblings, and extended families. Early adolescents’ mothers, aunts, and grandmothers were one of the most pervasive influences on their healthy eating and, to a lesser degree, physical activity. Early adolescents most frequently referenced their mothers, aunts, and grandmothers as promoting their physical activity by emphasizing the importance of weight loss and providing transportation to activities. However, when asked who made it easier for them to be physically active and eat healthy foods, all early adolescents cited their mothers.

When asked about people who made it easier for them to eat healthy foods, early adolescents also cited their grandmothers as important facilitators of their healthy eating. The early adolescents often preferred to eat at their grandmother’s house. They reported that they were more likely to eat healthy foods at their grandmother’s house instead of the school and community organization because they preferred their grandmother’s cooking.

Early adolescents’ schedules at home negatively influenced their healthy eating habits. They reported eating “a lot” of junk foods at home after school and on the weekends. A few early adolescents stated that their mothers often could not prepare dinner at night because of time constraints related to being too tired, watching television, caring for the children, or working.

Although families facilitated early adolescents’ physical activity at home, personal responsibilities sometimes acted as a barrier to physical activity. Family gatherings in which physical activities such as games were featured promoted early adolescents’ physical activities. Also, early adolescents often commented that they participated in sports to follow in the footsteps of one or more family members. However, early adolescents’ physical activities at home during the week were limited by their after-school responsibilities.

### Connecting with the community

*Connecting with the Community* refers to early adolescents’ beliefs and behaviours regarding ways they engaged with their community. Early adolescents’ perception of their community included their family members’ homes, school, and the community organization. Also, their weekly activities were limited to their home, school, and the community organization. Their daily interactions with the community were controlled by their parents or guardians. Early adolescents described attending family gatherings on the weekends at their family members’ homes. They also frequented local restaurants, retail stores, parks, and the other local community centres on the weekends and, less frequently, after school.

As evidenced by the large number of fast-food restaurants in the community and the lack of large supermarkets with affordable healthy food choices, the community environment did not support healthy eating. One of the main barriers to healthy eating that early adolescents reported was the availability of unhealthy foods within their homes and at the local grocery and convenience stores as well as a constrained budget that did not always allow purchasing the healthiest foods. Although early adolescents recognized the disadvantages of unhealthy eating, they noted that they often did not eat healthy foods when unhealthy foods were readily available.

Early adolescents described their preferred physical activities in the community. Many reported that they enjoyed what they referred to as play as their preferred unstructured physical activity. They would choose play that involved their peers and home when they were free to select how they spent their time. Many early adolescents also reported that they enjoyed playing basketball, football, and softball in the community parks or recreational centre.

Early adolescents described their school as helping facilitate both their physical activity and healthy eating through organized sports and physical education, health education, and weightlifting classes. Additionally, they identified their teachers and coaches at school as resources for their physical activity and healthy eating knowledge. Organized sports, especially basketball and football, were the main facilitators of physical activity for male early adolescents at school. However, not all male and female early adolescents qualified for organized sports teams. Furthermore, if they were not signed up for physical education or weightlifting classes at school, many early adolescents were sedentary for the majority of the school day.

Early adolescents identified connections between physical activity in school and in the community organization. In the community organization, many early adolescents’ physical activities were facilitated by their anticipation of participation in organized sports at school, even early adolescents who were not likely to qualify for organized sports teams at school. The community organization provided early adolescents with alternative opportunities for physical activity in the form of organized sports teams that did not exclude members. That is, there were no try-outs; any of the community organization members who wanted to participate could do so. Furthermore, the organized sports teams at the community organization functioned as practice for members who either anticipated trying out for organized sports at school or were unsuccessful in their first attempt at doing so.

### Peer influences

*Peer Influences* refers to influences that early adolescents perceived their peers had on their physical activity and healthy eating beliefs and behaviours. In this study, early adolescents described their peers as their friends, other early adolescents of the same age, siblings, and cousins. They also described the significance of their peers’ beliefs regarding physical activity and healthy eating.

Peers had both a positive and a negative influence on early adolescents’ healthy eating. For example, five early adolescents reported that they were more likely to eat healthy foods when with their peers who thought healthy eating was important, and seven early adolescents reported that their peers who preferred unhealthy foods had a negative influence on their healthy eating. Additionally, peers of early adolescents were observed encouraging unhealthy food consumption during snack time at the community organization.

Early adolescents frequently used the terms fun and play in their responses to interview questions. They used the term fun to describe enjoyable physical activity and educational experiences that were implemented through humour. They used the terms fun and play to describe physical activity that incorporated games and/or outdoor activities. Early adolescents described enjoying types of physical activities that included teamwork such as organized sports, especially basketball, and dance. In fact, early adolescents were observed laughing and smiling while participating in physical activities with their friends at the community organization.

Early adolescents talked about how their peers positively influenced their physical activities by encouraging play and participation in organized sports. They emphasized the importance of having playmates to encourage their physical activity, and many identified their siblings and cousins as their primary playmates at home. On the other hand, reported barriers to engaging in physical activities with their peers included lack of playmates and the need for parental permission to play with their peers.

### Developing a sense of self

*Developing a Sense of Self* refers to early adolescents’ descriptions of how their physical activity and healthy eating beliefs and behaviours were influenced by their developmental need of becoming an independent individual during early adolescence. In discussions of their self-development, early adolescents provided information that conveyed the importance of autonomy, recognition and respect, personal growth, and sensory perception on their physical activity and healthy eating behaviours. Early adolescents’ quest for self-development influenced their physical activity and healthy eating beliefs and behaviours in their interactions with their peers and families in their home, school, and community organization.

Early adolescents’ participation in physical activity was facilitated by their need for autonomy. Five early adolescents proudly stated that “no one” controlled their physical activity and that they could initiate physical activity independently anytime. At the same time, outside motivation was an important factor for early adolescents. One early adolescent stated, “if I feel inspired or somebody encourages me then I can do it. But if I encourage myself, then it won’t help that much, but I can control how much I exercise or do physical activity.”

Early adolescents were highly motivated to initiate and continue physical activity when provided with the opportunity to select their physical activity. They were observed expressing frustration when their teachers at the school and community organization forced them to participate in physical activities that the school or community organization selected on their behalf. Also, several early adolescents specifically reported that they felt more motivated to participate in physical activities when they were rewarded or felt respected.

Early adolescents’ physical activities (e.g., basketball and dancing on the sidelines) were oftentimes focused on the need to be recognized and respected by their teachers and coaches at the community organization and school and by their adult family members, peers, and siblings of similar ages. For example, as early adolescents danced or played basketball at the community organization, they often looked to see if others were watching them. Everyday when the researcher was at the community organization, female early adolescents were observed using a laptop computer to select songs for dancing on the sidelines of the basketball game.

Early adolescents thought that their current physical activity promoted their growth later in life, which was an important component of their self-development. They emphasized the importance of healthy eating to promote their mental functioning, which they identified as an important aspect of personal growth. However, early adolescents also associated healthy eating with promoting their physical health and strength. Male early adolescents felt strongly about playing basketball, which was conveyed in their discussions about basketball and in observations of their attentiveness while playing basketball at the community organization.

Early adolescents discussed developing their personalized and independent sensory perception of healthy foods. Many felt their sensory perception of certain nutritious foods (e.g., vegetables) was a barrier to healthy eating. They described the taste, visual appearance, smell, texture, and temperature as affecting their healthy eating behaviours. All early adolescents preferred the sweet, salty, and or spicy flavours as well as the “juiciness” of healthy foods. Similarly, early adolescents described their affinity for unhealthy junk foods as being associated with taste. Several early adolescents stated that they would not eat certain healthy foods that were not visually appealing, such as “plain looking” salad, “yucky looking” squash, “tree” like broccoli, and broccoli and Brussels sprouts appearing as if they were “just picked out of the garden.”

Early adolescents’ mothers, grandmothers, and aunts were important in the development of their individualized sense of taste for healthy foods. For example, early adolescents preferred the taste of foods prepared in unique ways by their mothers, such as squash with American cheese topping, creamed corn with butter, vegetables with hot sauce, green beans with baked chicken and salt to increase flavours of “sweetness” and juiciness, and greens beans and cabbage cooked with bacon.

## Discussion

We used an ethnographic approach to examine 15 early adolescents’ physical activity and healthy eating beliefs and behaviours. Semi-structured interviews and observations with these early adolescents at a community organization as well as artefact collection allowed for a deeper understanding of their beliefs and behaviours.

Our finding that early adolescents recognized benefits of physical activity and healthy eating supports the work of other qualitative researchers who identified that adolescents recognized health benefits such as maintaining a healthy body weight and physical appearance (Martins et al., 2015; Partida et al., 2018; Riggs et al., 2013). However, participants in our study emphasized unique benefits of both physical activity and healthy eating, such as increased bone health and immune functioning. More importantly, what this study adds to the existing body of research is the importance of a community organization in influencing early adolescents’ knowledge of the benefits and importance of physical activity and healthy eating. All early adolescents in our study attended a community organization that provided them with structured physical activity for at least one hour per day, healthy eating during snack time and dinner, and health promotion education.

Early adolescents identified their mothers, aunts, and grandmothers as one of the most pervasive positive influences of their healthy eating and, to a lesser degree, physical activity beliefs and behaviours. These findings are supported by other researchers who have identified the family parental unit as a key determinant of health for obesity prevention, physical activity, and health promotion in adolescents and children ranging in age from 6 to 18 years (Berge et al., 2012; Boutelle et al., 2012; Christiansen et al., 2013; McIntosh et al., 2010; Partida et al., 2018). However, in this study, the early adolescents specifically described their mothers, aunts, and grandmothers as promoting their physical activity by emphasizing the importance of weight loss and providing transportation to physical activity events. Moreover, when asked who made it easier for them to be physically active and eat healthy foods, all early adolescents cited their mothers.

Early adolescents’ perception of their community focused on their family members’ homes, school, and the community organization. It was here that their unstructured play and their participation in organized sports took place. Their families facilitated their physical activities in many ways. However, as seen in other studies, early adolescents’ physical activities in this study were limited by their responsibilities at home such as homework and taking care of siblings (Hannay et al., 2013; Swanson et al., 2013; Tuagalu, 2011).

Early adolescents identified their school teachers and coaches as well as their organized sports and physical and health education classes as facilitators of their physical activity and healthy eating. This is consistent with other studies, which have identified the importance of schools as an environment for educating and promoting physical activity and healthy eating (Bucher Della Torre et al., 2010; Wong et al., 2012). However, this study adds the early adolescents’ perspectives of how the community organization enhanced the physical activity and healthy eating benefits of the school. For example, all early adolescents had the opportunity to participate in sports activities at the community organization. They did not have to qualify for an organized sports team as they did in school.

Although the early adolescents had the benefit of a daily healthy snack and meal at the community organization, one of the main barriers to their healthy eating was the availability of unhealthy foods at the local grocery and convenience stores in the community. They noted that they were not as likely to eat healthy foods when unhealthy foods were readily available. Similarly, other researchers have noted the negative impact of the availability of unhealthy foods in the community on adolescents’ healthy eating (Caspi et al., 2012; Fleischhacker et al., 2011; Heidelberger & Smith, 2015).

Early adolescents discussed both positive and negative influences of their peers on their healthy eating. Other qualitative researchers have identified the influence of peer relationships on physical activity and healthy eating (Bailey-Davis et al., 2013; Christiansen et al., 2013; Krølner et al., 2011; Verstraeten et al., 2014; Wong et al., 2012). Additionally, a systematic review identified strong associations between peer interaction based on the closeness of interaction, type of physical activity, and gender (Chung et al., 2017). Early adolescents in this study talked about how their peers (i.e., “friends, other early adolescents of the same age, siblings, and cousins”) positively influenced their physical activities by encouraging play and participation in organized sports. Although some early adolescents reported a positive influence of their peers on their healthy eating, others reported that their peers’ preference for unhealthy foods had a negative influence on their healthy eating. Additionally, early adolescents were observed encouraging their peers to eat unhealthy snacks at the community organization. Similarly, Christiansen et al. (2013) found that adolescents aged 10 to14 years purchased the same unhealthy foods at the same locations as their peers.

Early adolescents frequently used the terms “fun” and “play” in their responses to interview questions. They used the term fun to describe enjoyable physical activity and educational experiences that involved humour. They used the terms fun and play to describe physical activities that incorporated games and/or outdoor activities. They described enjoying types of physical activity that included teamwork such as organized sports, especially basketball and dance, and they were observed laughing and smiling while participating in physical activities with their friends at the community organization. These findings support those of Berge et al. (2012) regarding the importance of fun as a facilitator of physical activity and healthy eating.

Many early adolescents identified the importance of having playmates such as their siblings and cousins to encourage their physical activity. At the same time, some early adolescents referenced a lack of playmates and lack of parental permission to play as barriers to their physical activities with peers. Similarly, other researchers have identified the lack of playmates and parental permission to go outdoors as barriers to outdoor play (Lee et al., 2015).

The early adolescents discussed their beliefs regarding autonomy, recognition and respect, personal growth, and sensory perception. They emphasized that it was important for them to establish or further develop control and autonomy over their physical activity and healthy eating behaviours. For example, they emphasized the importance of giving and receiving respect and being recognized while playing basketball and participating in other team activities. Similarly, Lee et al. (2015) found that it was important to give early adolescents a “voice” in choosing their preferred types of play to promote their physical activities, and Hingle et al. (2013) found that it was important to eliminate authoritarian tones when promoting and modifying physical activity and healthy eating behaviours in adolescents 12–18 years of age.

Early adolescents discussed the influences of taste, visual appearance, smell, texture, and temperature on their sensory perception and consumption of healthy foods. For example, many early adolescents determined whether they would eat fruits and vegetables based on their visual appearance. One early adolescent would not eat a vegetable because it looked “yucky,” while another early adolescent preferred to eat fruits that were her favourite colour. Similarly, other researchers found that taste influences healthy eating behaviours in adolescents (Callaghan et al., 2010; Karimi-Shahanjarini et al., 2010), and some found that the visual appearance of healthy foods influenced early adolescents’ decision whether to eat them (He et al., 2012; Krølner et al., 2011; Potter et al., 2011).

A strength of this study was that qualitative methods were used to explore both physical activity and healthy eating beliefs and behaviours in early adolescents in the context of a community organization. No other ethnographic studies were identified that explored both healthy eating and physical activity with a focus on a community organization in a racially underrepresented early adolescent population. Throughout the study, early adolescents frequently described the importance of the community organization as a facilitator of their healthy eating and physical activity. Additionally, healthy eating and physical activity seemed to be interconnected for the early adolescents. For example, as they responded about eating healthy foods, they connected their response to the importance of eating healthy foods for participating in organized sports or other physical activities. Furthermore, by exploring these two concepts together, we were able to gain insight into how the early adolescents were being taught about physical activity and healthy eating together in health promotion programs in the school and at the community organization.

This study was limited in the following ways. First, only early adolescents were interviewed, and they were only observed at one community organization. Interviews with the director and staff of the community organization as well as early adolescents’ schoolteachers, school staff, and parents would have provided additional perspectives about the early adolescents’ beliefs and behaviours. Observations of the early adolescents at home or in other community settings also may have provided additional insights. Second, the sampling strategy of recruiting early adolescents attending one community organization did not allow for comparison of the beliefs and behaviours of early adolescents with different socioeconomic backgrounds or ethnicity.

## Conclusions

The findings in this study suggest three different major influencers—family, peers and community organizations—that healthcare providers should consider when developing health promotion strategies to enhance early adolescents’ physical activity and healthy eating. Early adolescents’ family, especially their mothers, were reported as one of the most positive influences on their healthy eating and physical activity. Peers were sometimes seen as a positive influence, but other times as more of a negative influence. Additional research is needed to better understand peer influence and to design effective peer role modelling interventions that will promote healthy eating and physical activity.

More research is also needed on the contributions of community organizations to the development of healthy eating and physical activity in early adolescents. The early adolescents in this study reported how their community organization was instrumental in helping them with their healthy eating and physical activity. For example, unlike exclusive team sports and physical education courses that were sometimes offered at school, the community organization regularly gave all early adolescents who were members the opportunity to engage in physical activities in a safe environment.

Moreover, early adolescents in this study described how their need to make their own decisions, gain respect and recognition from their peers and other individuals, and develop their own palate for healthy foods influenced their physical activity and healthy eating behaviours. This suggests that the desire for autonomy is an important motivational force for early adolescents and should be taken into account when planning interventions to promote their healthy eating and physical activity.
